# The Impact of the SARS-CoV-2 Pandemic on Mental Health in Vulnerable Population Settings: The Case of Jordan

**DOI:** 10.3389/fpsyt.2021.692541

**Published:** 2021-07-22

**Authors:** Saverio Bellizzi, Lora Alsawalha, Luma Samawi, Ala'a Al-Shaikh, Hadeel Alfar, Nazeema Muthu, Maria Cristina Profili

**Affiliations:** World Health Organization, Jordan Country Office, Amman, Jordan

**Keywords:** COVID-19, mental health, refugees, migrants, Jordan, vulnerable population

## Introduction

The COVID-19 pandemic has negatively affected people's mental health across the globe and created new barriers for people already suffering from mental illness. According to a recent meta-analysis conducted on the impact of mitigation strategies on common mental health disorders, mental health concerns should not be viewed only as a delayed consequence of the COVID-19 pandemic, but also as a concurrent epidemic (1).

To make things more complicated, the pandemic has halted or at least disrupted critical mental health services in more than 90 percent of 130 countries in the world that has been proven from a participatory survey conducted by the World Health Organization (WHO) (2).

Over half of the WHO member state countries globally has reported disruption of access to services for vulnerable people, including children and adolescents (72%), the elderly (70%), and women requiring antenatal or postnatal care (61%). Additionally, around 75 percent of countries participated in above mentioned survey has reported at least partial disruption of workplace and school mental health services (2).

A very similar survey conducted by the WHO Eastern Mediterranean Region (EMR) Office showed higher level of disruption of services for mental health, neurological and substance use (MNS) disorders in countries in the region compared with global figures. The most disrupted were community/outreach services for people with MNS disorders (88.9%), psychotherapy/counseling/psychosocial interventions (85%), school mental health programmes (83.3%), services for children and older adults with mental health conditions or disabilities (83.3%) and work-related mental health programmes (73.3%) (3).

To note how the disruption of services like maternal health further compounded the mental health situation at country level; postpartum period specifically can seriously affect mental health of mothers in the absence of medical supervision (4).

## Vulnerable Populations in Jordan

Humanitarian emergencies in the Eastern Mediterranean Region have severely impacted human well-being, including the mental health of displaced populations and host communities. As far as Jordan is concerned, the protracted crisis in Syria coupled with the ongoing stressors related to displacement have had a significant impact on the mental health and psychosocial well-being of Syrian refugees as well as vulnerable Jordanians. In addition, such an influx of refugees from Syria has compounded already existing challenges resulting from hosting refugees from other neighboring countries, including Iraqis, Palestinians, Somalis and Yemenis (5).

## Mental Health Situation in Jordan

Since 2011, several surveys have been conducted to assess mental health conditions and needs for refugees and vulnerable hosting populations in Jordan. The 2013 WHO and International Medical Corps (IMC) assessment indicated high rates of mental health symptoms (present “most of the time” and “all of the time” in the last 2 weeks), with 53.9% and 49.4% of camp and non-camp respondents expressing feelings of anger and loss of control (6). Subsequently, a systematic review published in 2018 by UNHCR reported a lack of access to services by some groups of refugees due to financial and structural obstacles. An absence of awareness of available mental health services, coupled with widespread stigma in the community, were demonstrated to be the major barriers to effective access of mental health services (7).

A study (June 2019) conducted by the IMC during the pre-pandemic period showed that the proportion of participants with distress was 43.4%; 38.9% among the host population, 57.0% among refugees in urban communities, and 23.0% among refugees in camp (*p* < 0.005). Feelings of helplessness, lack of financial means, unawareness and poor recognition of mental health problems, cost of treatment, the need for privacy, and stigma were the primary obstacles to seeking help (8).

There are 46 Million professional and low-income labor migrants in eastern Mediterranean region. COVID-19 pandemic has exerted its toll on these migrant population, exacerbating threats to migrants' mental and psychosocial health, as many have now lost their jobs and are unable to provide for themselves or their families back home (9). Jordan is not an exception; of having 1.2 Million irregular migrants who relayed on daily wages. Loss of jobs and reduction of wage level were commonly occurring to these migrants creating an increased risk of mental health problems (10).

The psychological effects of quarantine and social distancing, isolation, loss of income and fear due to the COVID-19 pandemic have exacerbated existing mental health conditions especially among women and children, and those exposed to violence and forcible displacement (11). Massad et al. (12) showed that four out of ten people revealed some degree of anxiety during quarantine in Jordan. The same report confirmed how gender is a robust factor associated with the extent of psychosocial stress. A significant increase of domestic violence was also highlighted by the United Nations Population Fund (UNFPA): almost 70% of Jordanian women and girls were victims of some sort of gender-based domestic violence during the COVID-19 epidemic (13). On the other hand, the Multi-sectoral Rapid Needs Assessment jointly conducted by WFP, UNICEF and UNHCR in Jordan during the epidemic showed that 41% of all respondents witnessed a negative impact on their children's well-being because of the COVID-19 crisis (14). This further worsens the already difficult conditions stated in previous reports whereby 85% of the Syrian refugee children live in poverty and half of them suffer from various forms of sleep problems including nightmares or bedwetting as a result of the distress of being refugees (15).

## Discussion

The World Health Organization has been closely supporting the Jordanian Ministry of Health for several years on assessing mental health and psychosocial support for both Jordanians and refugees. The current COVID-19 Pandemic offers a unique opportunity to elevate the National Mental Health and Substance Use Action Plan 2018–2021, which was conceived around four pillars, each one with specific recommendations: (a) Governance, through an operational mental health Directorate guided and supported by the National Technical Committee; (b) Health care, through transition from institutional to community-oriented integrated model of care as well as from biomedical to bio-psychological care; (c) promotion and prevention, through development of programmes such as suicide prevention programme, parent skills training for children, and targeted mental health literacy programme to reduce stigma and discrimination; (c) surveillance, monitoring and research, through regular monitoring of quality of services and incorporation of limited categories of mental disorders in national information systems.

The Jordan response actions to the current epidemic were not really integrated within existing services for mental health to a sufficient extent, and there remains a need to train non-specialists to be able to respond to mental health needs in the population in an emergency setting using tools such as Psychological First Aid, and mhGAP-Humanitarian Intervention Guide (mhGAP-HIG) and Problem Management Plus (PM+) just to name a few.

World Health Organization and International Labor Organization launched an appeal on 26 July 2020 for Eastern Mediterranean Region, requesting member states to offer universal health coverage for migrants and to have voluntary access to testing, isolation and treatment, with full respect for their dignity, human rights and fundamental freedom. Both organizations advocated for continuity of essential services to displaced populations and migrants, including mental health and psychosocial support and the management of non-communicable diseases, in addition to gender-based violence (9).

Strengthening the national mental health system is an integral part of health system management and sustainability, given that, it is staffed by the appropriate mix of trained workforce and for mental health to receive a specified budget to implement the national action plan through MOH.

Disruption of mental health services should be monitored and promptly addressed in a systemic manner and in line with response and recovery plans along with the other components related to maintaining essential health services. Integration of such aspects in national plans and their financial aspects should be carefully taken into consideration: despite the fact 89% countries reported that mental health was part of their COVID-19 response plans, only 17% revealed full additional funding for covering these activities (2).

Establishing a mental health and psycho-social strategy is of critical importance: inclusion of targeted actions toward COVID-19 cases, contacts, family members, frontline workers and the broader community, with special attention to the needs of vulnerable groups is needed to address needs in a holistic way; this would also include addressing mental health and basic needs of people with pre-existing mental health conditions who are affected by COVID-19, addressing stigma, and integrating response activities into existing services (16, 17).

As highlighted by Javed et al., the mental health effects of COVID-19 are as important as understanding clinical and epidemiological aspects of the disease itself (18). This becomes even more important in contexts like Jordan where the large presence of vulnerable populations such as refugees, migrants and vulnerable host population requires tailored and integrated strategy. Such strategies should lead to enhanced community awareness on how to maintain mental health is critical.

## Author Contributions

SB, LS, HA, and MP conceived the idea. LA, NM, and AA-S conducted the literature review. SB, LS, NM, and AA-S wrote the first draft. All authors revised and finalized the manuscript.

## Conflict of Interest

The authors declare that the research was conducted in the absence of any commercial or financial relationships that could be construed as a potential conflict of interest.
